# Schistosomiasis in pre-school-age children and their mothers in Chikhwawa district, Malawi with notes on characterization of schistosomes and snails

**DOI:** 10.1186/1756-3305-7-153

**Published:** 2014-04-01

**Authors:** Helen Poole, Dianne J Terlouw, Andrew Naunje, Kondwani Mzembe, Michelle Stanton, Martha Betson, David G Lalloo, J Russell Stothard

**Affiliations:** 1 Department of Parasitology, Liverpool School of Tropical Medicine, Pembroke Place, Liverpool L3 5QA, UK; 2 Department of Clinical Sciences, Liverpool School of Tropical Medicine, Pembroke Place, Liverpool L3 5QA, UK; 3 Malawi-Liverpool-Wellcome Trust Clinical Research Programme, PO Box 30096, Chichiri, Blantyre 3, Malawi

**Keywords:** Preventive chemotherapy, Praziquantel, *Schistosoma haematobium*, *Schistosoma mansoni*, SEA-ELISA, Zoonosis

## Abstract

**Background:**

To complement ongoing schistosomiasis control within national control programmes (NCPs) that administer praziquantel to school-age children, assessing the risk and extent of schistosomiasis in pre-school-age children (PSAC) is important.

**Methods:**

In June 2012, schistosomiasis in Chikhwawa district, Malawi was assessed across 12 villages examining pre-school-age children (PSAC) and their mothers by serological and parasitological diagnosis, as supplemented with urine-antigen and questionnaire-interview methods. Urinary tract morbidity was inferred by haematuria and albuminuria assays.

**Results:**

In total, 49.5% (CI_95_ 42.6-56.4) of 208 PSAC and 94.5% (CI_95_ 90.9-98.1) of 165 mothers were seropositive for schistosomiasis, in 2 villages seroprevalence exceeded 75% in PSAC. Egg-patent urogenital and intestinal schistosomiasis was observed; 17.7% (CI_95_ 12.4-23.2) of PSAC and 45.1% (CI_95_ 37.4-52.8) of mothers having active schistosomiasis by parasitological and urine-antigen testing combined. PSAC often had extensive daily water contact and many (~25%) had haematuria and albuminuria. As eggs with an atypical morphology of *Schistosoma haematobium* were observed, a general selection of schistosome eggs was characterized by DNA barcoding, finding Group I *S. haematobium* and Group IV and V *S. mansoni*. Malacological surveys encountered several populations of *Bulinus globosus* but failed to find *Biomphalaria.*

**Conclusions:**

Both PSAC and their mothers appear to be at significant risk of schistosomiasis and should be considered for treatment within the NCP of Malawi.

## Background

Two major forms of schistosomiasis exist in sub-Saharan Africa (SSA), urogenital and intestinal, each caused by infection with different schistosome species, *Schistosoma haematobium* and *S. mansoni*, respectively [1]. In order to complete their lifecycles, schistosomes require aquatic intermediate snail hosts, thus the distribution of susceptible populations of *Bulinus* and *Biomphalaria* broadly outlines the endemic areas where urogenital and intestinal schistosomiasis occur [2]. Surveying local freshwater habitats for such snails is particularly useful for assessing transmission risk [3,4] and often allowing differentiation between autochthonous or imported infections as recently shown for intestinal schistosomiasis on the Sesse Islands, Uganda or urogenital schistosomiasis on Mafia Island, Tanzania [5,6].

Whilst co-infection of both types of schistosomiasis is known, its extent remains poorly quantified but it is likely that several tens of millions live with both forms of disease [7] which may have permitted some ancestral genetic introgression between species previously [8]. In addition, there is now growing evidence for zoonotic transmission of urogenital schistosomiasis, so surveillance systems should become increasing alert to this possibility [9]. Thus where sanitation and water hygiene is poor, up to 800 million people are at risk of schistosomiasis and this is often coupled with low levels of disease awareness among afflicted communities [10,11]. Preventive chemotherapy with praziquantel (PZQ) is the main control strategy [12]. With international support [7], several national control programmes (NCPs), including Malawi, are active in conducting mass drug administration (MDA) of PZQ. Indeed access to PZQ has expanded vastly in recent years [1,7,12] and in line with the WHO Strategic Plan for Control of Schistosomiasis, in the forthcoming 2012-2020 period, further scale-up of MDA is predicted in SSA with up to 250 million tablets earmarked each year for treatment of school-aged children (SAC) alone [11].

In highly endemic areas, however, members of the community other than SAC can be infected. Such groups are often overlooked in terms of their treatment needs [13-15]. For example, recent attention has focused upon documenting prevalence of infection in pre-school-age children (PSAC) and in so doing has defined a clear ‘PZQ treatment gap’, i.e., PSAC in need of treatment are generally excluded from MDA programmes [14,16,17]. The reasons behind this gap are complex but include the absence of a suitable PZQ pediatric formulation. The WHO has now recognized that where a need is shown, infected PSAC should be provided with crushed or broken PZQ tablets [11,15,18,19], as a pragmatic stop-gap [13,20-22], until an appropriate pediatric PZQ formulation becomes available.

To better estimate disease, it remains important to collect up-to-date epidemiological information to inform treatment strategies generally [23,24] and specifically that needed for PSAC in the context of present and future control [13]. Diagnosis of schistosomiasis in young children is problematic as there is no ‘gold-standard’ [14,23]. The detection of infections in PSAC is somewhat different to older children as the adult worm pairs are themselves maturing into full fecundity, so egg-detection methods perform poorly having a significant time-delay lagging behind the patency of either antibody or antigen methods [25]. Serological analysis by detection of host antibodies to schistosome soluble egg antigen (SEA) is recognized as the most sensitive method of detection but cannot differentiate between the different forms of schistosomiasis or identify co-infection [25,26], while egg-detection methods, which are generally acknowledged to lack sensitivity, have high specificity for each type of schistosomiasis [11]. In Uganda, for example, using a combined diagnostic approach of host serology (i.e., SEA-ELISA for IgM/G antibodies) and urine-antigen rapid diagnostic tests (i.e., urine immuno-chromatographic dipsticks for circulating cathodic antigen (CCA)) demonstrated that over 50% of PSAC had intestinal schistosomiasis [25,27]. Many of these children also had overt morbidity, including organomegaly, anemia and stools positive in faecal occult blood [28,29]. In Cote d’Ivoire, Niger, Nigeria and Mali, urogenital schistosomiasis can be common in PSAC [15,30,31]. Putative morbidity can be assessed by ultrasonography and by POC assays for haematuria and albuminuria, used alongside detection of intestinal schistosomiasis with urine-CCA dipsticks [7,32-34].

In Malawi the schistosomiasis NCP focuses upon treatment of SAC and the epidemiology of schistosomiasis, albeit urogenital or intestinal forms, in PSAC is presently unclear. Here we report on a pilot investigation of schistosomiasis in PSAC and their mothers in an area around Chikhwawa town, located in the Lower Shire River valley. The epidemiological survey was structured to address, as best possible, infections within PSAC using a combination of diagnostic methods with serological and parasitological methods supplemented with urine-antigen and questionnaire-interview methods. To estimate levels of urinary tract disease, assays for detection of haematuria and albuminuria were applied. To further investigate aspects of the local epidemiology of schistosomiasis, molecular characterization of encountered schistosomes was undertaken alongside malacological surveys to estimate environmental risk of transmission.

## Methods

### Study area and participants

Chikhwawa district is located within the southern lowland region of Malawi (16° 1′ S; 34° 47′ E), an area characterized by a tropical climate with mean annual temperature of 26°C, a single wet season from November to April, and annual rainfall of approximately 770 mm (Malawi Meteorological Office, Chileka, Blantyre). The Lower Shire River empties directly from Lake Malawi and flows in a Southern direction. Flooding and the development of water bodies up to several kilometers away from the river are common following the rainy season. Major local agricultural projects also have extensive irrigation systems, e.g. at the Nchalo sugar cane estate. There are large roving herds of cattle, however, the extent of bovine schistosomiasis, or its zoonotic potential, is unknown.

**Figure 1:**
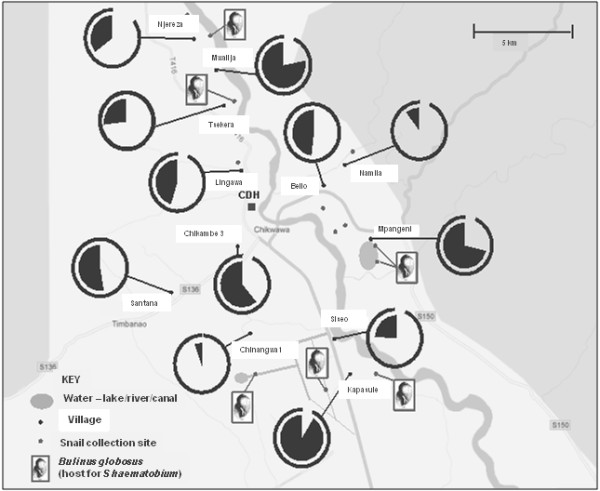
A sketch map of snail collecting sites around Chikhwawa showing the 12 villages surveyed for schistosomiasis as indicated by SEA-ELISA by pie-charts denoting prevalence in PSAC (black pie segment infected) and in mothers (outer line segment infected).

A continuous Malaria Indicator Survey assessing the year round intervention coverage and disease burden was ongoing in children aged 6-59 months and adults from over 50 villages within Chikhwawa [35,36]. In July 2012 a survey for schistosomiasis in PSAC was conducted alongside this survey in 12 villages that represent varied habitats within the local area, see Figure 1. At the point of survey, GPS co-ordinates in decimal degrees were taken using a handheld *e-trex* unit (Garmin, Southampton, UK) to enable later cartography. In line with rapid survey methods for schistosomiasis [11], a target of 15 consenting mothers each with a child aged between 6 months to 5 years from within the sampled village were invited to participate in a semi-random sample; if a mother was selected and had more than one child within this age range, then her other children were also included.

### Specimen collection, diagnostic procedures and PZQ treatment

As parasitological methods are known to be insensitive, host serology was considered to be most informative in the context of the PSAC as a single serum sample can be obtained directly with minimal discomfort and there are now commercially available ELISA kits that can be used in resource poor laboratories [25]. Between 4 to 5 drops of fingerprick blood were collected from each participant, allowed to clot in a 1.5 ml eppendorf tube and stored a cool box until later centrifugation at 6,000 rpm for 10 minutes in a portable micro-centrifuge (Galaxy Ministar, VWR, Pennsylvania, USA). A total of 2.5 μl sera was used in a commercially available SEA-ELISA kit, *Schistosoma* Antibody Detection Test (SCIMEDX, New Jersey, USA). To ensure consistency, positive and negative controls were loaded onto each ELISA plate at the beginning and end of loading of sera samples [25]. ELISA plates were processed and incubated according to manufacturer’s protocols. Upon completion, reaction wells were examined visually against a white card background, by 2 people independently. Reactions were graded as strong positive (+++), medium positive (++), light positive (+), trace (tr) and negative (-) using a standard photograph of wells demonstrating this range from dark yellow to colourless.

Owing to logistic constraints in terms of local technical microscopy support and an anticipated low endemicity of *S. mansoni* and soil-transmitted helminthiasis (STH), parasitological examination of faecal material was performed in the first 6 villages only. In these 6 villages, 100 ml plastic containers were distributed to adult and child participants the evening before the day of survey for collection of a morning stool specimen. Urine containers were distributed to participants in all 12 villages for collection of a mid-morning sample from each mother and her child/children on the day of survey. A single thick Kato-Katz (41.7 mg) smear was prepared from stool samples on the day of collection to detect intestinal schistosomiasis and STH [25]. This was examined under microscopy (x100), by two independent slide readers, for the presence of helminth eggs. Eggs were counted, tabulated, expressed as mean eggs per gram (epg) of faeces and then classified according to WHO egg intensity guidelines, e.g. for intestinal schistosomiasis of light (< 100 epg), medium (≥100 & < 400) and heavy (≥ 400 epg) categories, for each species of infecting helminth [11]. To increase the sensitivity of urine filtration to detect urogenital schistosomiasis, 20 ml of urine was filtered by plastic syringe through a 13 mm diameter cut circular nylon filter of 35 μm pore-size. Filters were then stained with a drop of Lugol’s iodine and viewed under microscopy (x100) counting eggs of *S. haematobium* to be later expressed as light (< 50 eggs/10 ml) or heavy (≥ 50 eggs/10 ml) infection intensities according to WHO guidelines [11].

All urine samples were tested for the presence of schistosome CCA on site using a lateral flow immuno-chromatographic urine dipstick RDT (Rapid Medical Diagnostics, Pretoria, South Africa). The urine CCA-dipstick is currently advocated as an alternative mapping tool to Kato-Katz for the detection of intestinal schistosomiasis and is not recommended for detection of urogenital schistosomiasis owing to very low sensitivity [25,26,37,38]. According to the visual staining intensity of the test band, results were classed as negative (-), trace (tr), positive (+), medium positive (++) or strong positive (+++). Visual trace results were considered negative in the analysis of results and any positive result was interpreted as indicative of an active infection with *S. mansoni*[37].

### Indirect measures of urinary tract morbidity and levels of anaemia

Documentation of putative pathology associated with urogenital schistosomiasis was assessed by proxy methods of urine analysis including: visual inspection, reagent strip analyses and POC urine-albumin assays. Anaemia was quantified by point-of-contact (POC) haemoglobin measurement. *Visual inspection* - all urine samples were visually inspected for colour and clarity using a colour chart and turbidity scale (lined barcode pattern) placed underneath the urine container. *Reagent strip analyses* - the presence of blood, protein, nitrates and leucocytes was estimated. POC *urine-albumin* - urine albumin was quantified using the Hemocue® albumin system (Hemocue, Sweden). Turbidity was removed from the urine sample by centrifuging 0.5 ml of each urine sample in a 0.5 ml Eppendorf tube for 5 minutes at 6000 rpm in a portable micro-centrifuge (Galaxy Ministar, VWR, Pennsylvania, USA) before a visual turbidity absorption reading was taken using the Hemocue® cuvette, expressing urine-albumin concentration from 5-150 mg/l. Samples above this range were diluted by 1:3 serial dilutions in normal saline as recommended by the manufacturer until a reading within range was obtained. *Anaemia* – a fingerprick blood sample was used to measure haemoglobin concentrations of mothers and children (Hemocue**®** Hb201+ system, Hemocue, Sweden). As Chikhwawa is below 150 m above sea level, altitude correction was not required.

### Case history questionnaire and recalled water contact patterns

Each mother was interviewed using a standardized questionnaire in the local language (Chichewa). Data were collected on socio-demographic factors (i.e., age, sex, educational level), knowledge and awareness of the disease, its basic symptoms, and water-use and water contact patterns. A copy of the questionnaire is available upon request from the corresponding author.

### Molecular characterization of schistosomes

In order to retrieve schistosome eggs for DNA barcoding, pooled stool or urine samples were obtained from infected children at Kapsasule, Mpangeni and Mwaliga (*see* Figure 1) and processed as previously decribed [39-41]. Eggs or hatched miracidia were harvested by pippette in 3 μl of water then placed onto FTA indicator cards. From the FTA punch material, a 956 bp region of the mitrochondrial cyctochrome oxidase subunit 1 (*cox*1) gene, or DNA barcode, was amplified for *S. haematobium* in separate 25 μl PCR reactions using illustraTM puReTaq Ready-To-Go PCR Beads (GE Healthcare, UK) and 10 pmol of each primer (Forward primer: COX1_Schisto_5′; Reverse primer; COX1_Schist_3) [40]. For *S. mansoni*, a 540 bp fragment was amplified or 3 μl of genomic DNA using the ASMIT1 and Cox1_Schist_3′ primers and illustra™ puReTaq Ready-To-Go PCR Beads (GE Healthcare) [39]. Amplification products were later sequenced in both forward and reverse directions for assembly as edited with Sequencher ver. 4.6 (http://www.genecodes.com). Sequences were then compared against those in EMBL/Genbank using the Basic Local Alignment Search Tool (BLAST) to ascertain which of the known groups of *S. haematobium* (Group I or II) [40] and *S. mansoni* (Group I, II, III, IV or V) would be assigned [41].

### Malacological surveys for freshwater snails

Prior to the parasitological surveys, each village was visited to inspect local freshwater habitats for freshwater snails. To find sites, a combination of GoogleEarth satellite imagery was used in conjunction with local knowledge of villagers and direct sighting of marshy areas/standing water bodies. Owing to local access opportunities, a total of 16 freshwater sites were inspected for snails using hand held metal scoops and direct picking with forceps. Temperature, water conductivity and pH were recorded at each site using a handheld meter (Hanna Instruments, UK). Direct observations of water contact of PSAC was also made, and recorded by photography, at the time of survey in or around the freshwater habitat. Sampling at each site was semi-quantified, with two collectors surveying each for approximately 20 minutes at each site. All collected snails were exposed to sunlight for several hours to check for infection with *Schistosoma* spp. or other parasite cercariae. Snail species were identified on the basis of shell morphology and for species within the *Bulinus africanus* group, by anatomical dissection and inspection of the genitalia [2].

### Ethical considerations and treatment

Ethical approval was granted from the College of Medicine Research Ethics Committee (COMREC, Blantyre, Malawi) and from the LSTM Research Ethics Committee (Liverpool, UK). Local support was received from the District Health Officer and village chiefs. After community village sensitization, written informed consent was obtained for women and children from each mother either as a signature or thumbprint.

Any mother or PSAC that was found infected by either urine-CCA dipsticks or upon evidence of haematuria by reagent strips was treated on site with PZQ (40 mg/kg). To facilitate accurate dosing, patient’s weight was measured for all participants using a digital weighing scale [19-21]. Younger children were provided with crushed tablets as mixed with orange syrup along with a local food item (biscuit) and potable water. After treatment, all children were monitored informally for up to 2-3 hours to monitor for immediate complications such as vomiting. Upon completion of SEA-ELISA and parasitological testing, a second visit to each village was undertaken to ensure that all positive participants received PZQ treatment.

### Data management and statistical analyses

Data were recorded onto paper-format questionnaires and results sheets using participants’ unique study numbers and double entered into Excel (Microsoft, USA). Discrepancies were resolved upon inspection of paper copy records. Univariate and multi-variate analyses were performed in SPSS (v. 20 IBM SPSS statistics 2011) to assess associations between infection status and putative risk factors from case-history questionnaire data collection (i.e. recalled water contact).

## Results

### Prevalence of schistosomiasis

**Figure 2:**
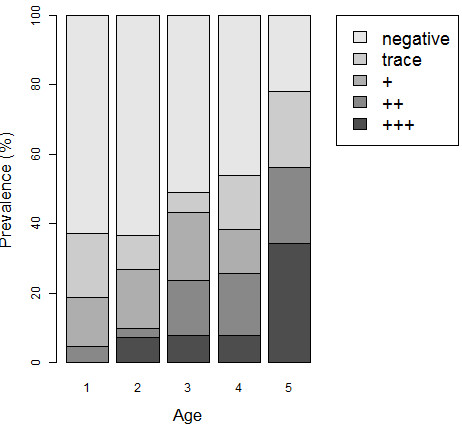
Observed prevalence of SEA-ELISA positive results in PSAC by age group and by strength of reaction.

165 mothers (mean age 28 years range 18-47) and 208 children (mean age 36 months range 9-66, gender ratio 1.1 boys:girls) participated in the study. 94.5% of mothers (CI_95_ 90.9-98.1) and 49.5% of PSAC (CI_95_ 42.6-56.4) were seropositive for schistosomiasis by SEA-ELISA. The geographical distribution of seropositive PSAC and mothers across the 12 villages is shown in relation to the main water bodies and where *Bulinus* snails were encountered (Figure 1). The seroprevalence varied widely from over 50% of PSAC in Kapasule, Mpangani, Mwalija, Chikambe 3 and Santana villages to less than 5% in Chinangwa 1 and Namila, indicating a focal distribution. Both the proportion of positive SEA-ELISAs and the titre increased with increasing age of children ranging from 37.2% (CI_95_ 22.1-52.3) in PSAC aged 1 to 78.1% (CI_95_ 62.9-93.2) in PSAC aged 5 years (Figure 2). There was no significant difference in prevalence between boys and girls. In total, 45.1% (CI_95_ 37.4-52.8) of mothers and 17.7% (CI_95_ 12.4-23.2) of children were found to be infected by parasitological (urine-filtration) and/or CCA-dipstick testing.

In the 6 villages where stool samples were examined, egg patent *S. mansoni* infection was observed in 21.5% (CI_95_ 12.3-30.7) of mothers but only one 5 year-old boy. In these 6 villages the urine CCA-dipstick was positive in 33.3% (CI_95_ 22.8-43.8) in mothers and 10.5% (CI_95_ 4.3-16.8) in children. The overall prevalence based on CCA dipsticks across all 12 villages was 24.9% for mothers (CI_95_18.2-31.5) and 9.1% for children (CI_95_ 5.1-13.2). No other helminth ovum was seen in the stool. Microscopy of filtered urine showed that 25.0% of mothers (CI_95_ 18.3-31.7) and 10.7% (CI_95_ 6.4-15.1) of children were excreting eggs of *S. haematobium*. Three mothers had atypical eggs of a terminal spined schistosome that were approximately twice the typical 80-90 μm length of *S. haematobium*. The highest egg-patent prevalence of urogenital schistosomiasis was found at Kapasule with 60.0% (CI_95_ 31.9-88.1) of mothers and 42.8% (CI_95_ 19.7-65.9) in PSAC indicative of a local hot-spot and consistent with SEA-ELISA findings.

**Figure 3:**
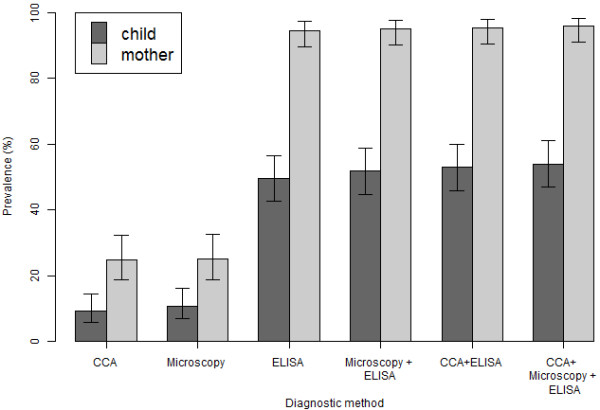
**Prevalence of schistosomiasis as assessed by different diagnostic methodologies in PSAC and mothers across the 12 villages [CI**_**95 **_**around the prevalence are indicated].**

Pooling of positive results from all three techniques (stool & urine examination, CCA-dipsticks and SEA-ELISA) demonstrated an overall prevalence of 53.9% (CI_95_ 47.0-60.9) in PSAC (Figure 3). As children are seronegative for schistosomiasis until they acquire their first infection, the SEA-ELISA can be considered the most sensitive method for detection of initial active infection but cannot differentiate between the two forms of schistosomiasis and might time-lag slightly behind urine-antigen methods. Urine-CCA dipsticks are considered to be excellent proxy markers of intestinal schistosomiasis and are not confounded in this instance by urogenital schistosomiasis. The prevalence of co-infection with both *S. haematobium* and *S. mansoni* was estimated to be 8.5% in mothers (CI_95_ 4.2-12.8) and 3.6% in children (CI_95_ 1.0-6.2) using criteria of a positive CCA-dipstick (*S. mansoni*) and urine-filtration/microhaematuria (*S. haematobium*).

### Indirect markers of morbidity

**Figure 4:**
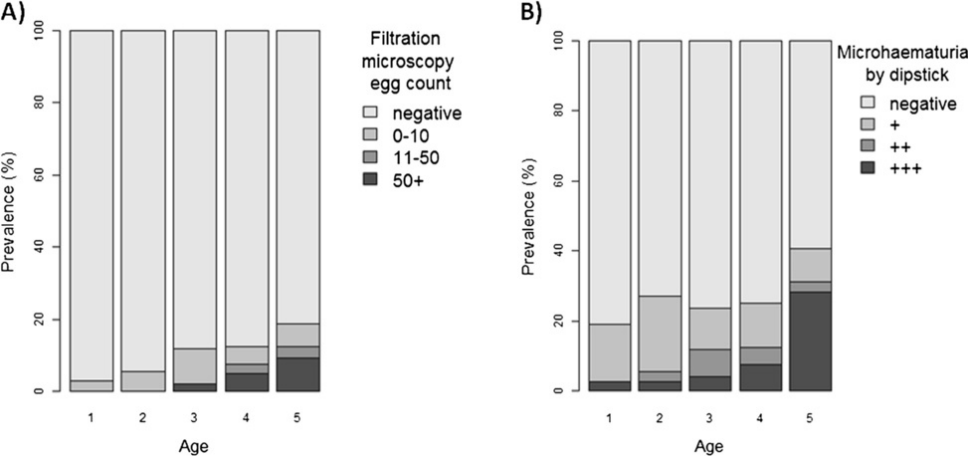
A & B Frequency of egg-patent urogenital schistosomiasis increases with age of the child (A), microhaematuria is also associated with increasing age (B).

**Figure 5:**
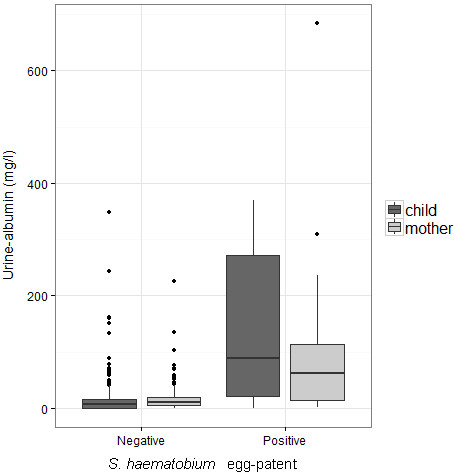
**Raised urine albumin levels (> 40 mg/L) in relation to egg-patent infection with *S. haematobium *** **reveal underlying urinary tract pathology in young children.**

38 mothers and 17 children had turbid urine upon visual inspection. This was significantly associated with egg patent *S. haematobium* infection in both mothers and children (OR 8.1 CI_95_ 4.2 – 15.3). Only 4 mothers and 2 children presented with macrohaematuria; all of these were egg-patent for *S. haematobium.* Reagent strip testing demonstrated microhaematuria in 45 women (40.0% [CI_95_ 32.5-47.5] and 57 children (24.4% [CI_95_18.3-30.4] haematuria). The presence of microhaematuria was significantly associated with egg patent *S. haematobium* infection in all participants (OR 15.5 CI_95_ 7.8 – 30.9) and clearly increased with child age, Figure 4A & B. Elevated urine albumin levels (>40 mg/l) were seen in 26.7% of mothers and 17.3% of children, with no significant difference between boys and girls. The mean levels of urine-albumin related to results of diagnostic methods for *S. haematobium* are shown in Table 1; significantly higher levels of urine-albumin were associated with infection with urogenital schistosomiasis. A box-plot of the relationship between urine-albumin and egg-patent infection with *S. haematobium* is shown in Figure 5*.*

**Table 1:** **Mean urine albumin values and relationship with parasitological, immunological and proxy marker (microhaematuria) of infection with *S. haematobium***

	**Mothers**		**Children**	
	**Mean urine albumin (mg/L)**	**CI**_**95**_	**Mean urine albumin (mg/L)**	**CI**_**95**_
All	11.9	9.1 – 15.4	7.6	5.9 – 9.8
*S. haematobium* egg-negative	7.4	5.8 – 9.8	5.7	4.4 – 7.5
*S. haematobium* egg-positive	43.3	28.6 – 65.5	49.0	20.1 – 117.4
SEA-ELISA negative	14.3	7.1 – 27.9	4.1	2.9 – 5.6
SEA-ELISA positive	11.7	8.9 – 15.3	13.9	9.2 – 20.7
No microhaematuria	6.3	4.6 – 8.5	4.6	3.4 – 6.1
With microhaematuria	27.2	18.8 – 39.2	27.1	16.1 – 45.1

Upon fingerprick blood testing, clinical anemia was present in 40.6% mothers (<120 g/L) and 71.9% of children (<110 g/L). The mean hemoglobin for mothers was 122.5 g/L (CI_95_ 120.1–125.0) and 101.1 g/L for children (CI_95_ 99.0-103.1). There was no statistically significant difference between hemoglobin levels in children in relation to sex or infection with schistosomiasis (using any diagnostic method).

### Case history questionnaire and recalled water contact patterns

The majority of mothers had received little or no formal education; 44% of the mothers had never attended school, 53% had attended at primary level and just 3% having attended secondary education. All mothers reported that the central district hospital (Chikhwawa) was where they took their children if they became unwell. General awareness of schistosomiasis was very poor, 97% of the women had little or no knowledge of the disease, despite a quarter of them verbally reporting to have had previous PZQ treatment.

All but one family had access to safe water via working boreholes in every village. However, 18% and 54% of mothers respectively would wash themselves or their clothes in environmental water (rivers, lakes or canals). Up to 20% of PSAC were reported to be bathed at least once (60%) or twice (38%) in this potentially contaminated water each day. 41% of PSAC were spending more than 30 minutes in or around the water margins each day. There were no statistically significant associations between egg-patent schistosomiasis and the potential risk factors examined in the questionnaire.

### DNA barcoding of schistosomes

A total of 25 *cox*1 barcodes were assembled from *S. haematobium* which all matched a previous GenBank entry EU567128 - Malawian *S. haematobium* Group I DNA barcode even though the *cox*1 was amplified from two atypical eggs. Owing to much less FTA card material, in total 5 *cox*1 barcodes were assembled from *S. mansoni* retrieved from Namila village. These sequences matched two previously known *cox*1 barcodes JQ289610 – *S. mansoni* Group IV (Coastal Kenya and Zambia) and JQ289739 – *S. mansoni* Group V (Zambia) and is the first time Malawian *S. mansoni* has been characterized at the molecular level.

### Malacological surveys

16 freshwater sites were inspected for the presence of aquatic snails, these habitats ranged from natural wet marshy areas adjacent to the Shire River to large ponds that represent permanent standing water bodies e.g. the ox-bow lake at Mpangeni, as well as artificial habitats such as irrigation scheme canals (Figure 1). Across these habitats water chemistry values ranged broadly: pH 8.4-9.5, conductivity 274-1505 μS and temperature 28.5-33.6°C. A total of 7 species of mollusc were encountered with certain species more broadly distributed across the habitats than others; 2 locations were devoid of snails. Following morphological identification keys, the species list with number of times encountered (n *sites encountered*) is as follows: *Bellamya* sp. (5), *Bulinus globosus* (7), *Bulinus forskali* (7), *Physa* sp. (2), *Lanistes* sp. (7), *Lymnaea natalensis* (2) and *Melanoides* sp. (8). No population of *Biomphalaria* was found nor remnant shells thereof. Whilst over 250 *B. globosus* were collected across the 7 habitats encountered, none was observed to shed fork-tailed cercariae. This snail species was most abundant at the ox-bow lake of Mpangeni.

## Discussion

Our survey has added to the growing body of evidence that PSAC can have overt schistosomiasis and has revealed the occurrence of both urogential and intestinal schistosomiasis in Chikhwawa. The occurrence and severity of schistosomiasis in PSAC is currently receiving considerable attention for two reasons. First, it is increasingly recognized that the extent and significance of disease in this age group has been largely overlooked [23]. While this is now being addressed by changes in generic WHO de-worming guidelines that include PSAC, this will inevitably lead to a considerable underestimation of the PZQ needed within the NCP [24]. PZQ is presently donated free to WHO then is later distributed to each NCP for preventive chemotherapy in SAC alone [13,14]. Furthermore, off-label use is not formally encouraged by the donor, MercK-KGa, owing to ambiguities in original licensing of PZQ (Biltricide®). To counter this, an initiative to develop an appropriate pediatric formulation with revised pharmaceutical licensing and labelling is ongoing [13]. Second, improved estimates of the burden of disease and need for treatment of PSAC within NCPs, and better understanding of the challenges of programme delivery in the group is urgently needed. Our findings also highlight the need to evaluate schistosomiasis in PSAC in other parts of Malawi where urogenital schistosomiasis is considered hyper-endemic and significant morbidity in other groups has been reported [42], for example, a recent survey in Zomba near Lake Chilwa reported an egg-patent prevalence of *S. haematobium* in 20% of the PSAC surveyed [43]. Identification and treatment of these children is needed, ideally with an appropriate pediatric PZQ formulation, but until then, crushed or divided tablets can be used [15,19,44-46].

### The extent of schistosomiasis

Accurately estimating the prevalence of schistosomiasis in PSAC is not easy especially when infections are only recently acquired and therefore egg burdens may not have reached diagnostic thresholds of all detection methods [14]. Similarly, host antibody responses, although very sensitive, can have a 2-3 month temporal lag behind schistosome antigen detection methods [25]. Nonetheless, the extent of schistosomiasis in PSAC was considerable, with slightly more than half (53.9%) having evidence of infection and although the relationship of serology and active infection can be confounded by history of treatment. In the context of PSAC, where there has been no previous treatment, then SEA-ELISA in this instance could be considered the ‘gold standard’. Increasing age was significantly associated with increased prevalence and intensity of antibody responses and egg-patent infections, in keeping with results from studies elsewhere [16]. This occurs both because the cumulative level and daily duration of exposure in environmental water bodies increases as the child grows older and because schistosome worm populations in infected children mature to full egg laying fecundity, thereby increasing SEA levels. The high levels of cumulative water contact in habitats where suitable snails were found in this study suggest continuous local risk of transmission in this age group and is similar to those previously demonstrated in PSAC on the shoreline of Lake Albert [47].

**Figure 6:**
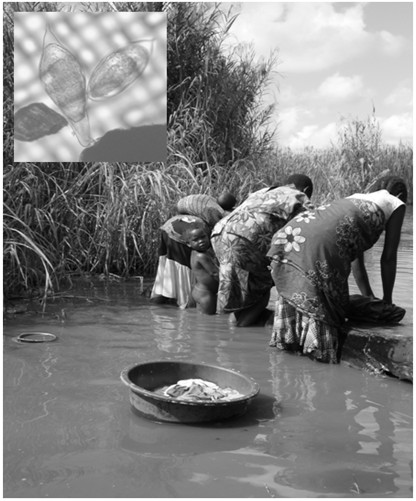
**PSAC frequently accompany their mothers into the water when washing upon concrete slabs as shown here at Mpangeni [Inset: an atypical egg (left) alongside a typical egg (right) of *S. haematobium.*** The egg on the left is approximately 190 μm in length and resembles *Schistosoma leiperi*, a schistosome commonly found in wild antelopes].

The situation in mothers is perhaps even more alarming as 94.5% of mothers were positive for schistosomiasis by SEA-ELISA with slightly under half (45.1%) having active infection upon the basis of egg excretion or CCA-dipstick results [22]. It is well-known that parasitological methods are insensitive for intestinal schistosomiasis which has led to the creation of a pocket-prevalence-chart to correct upwardly observed egg-based prevalence values [48] and using this chart would infer a ‘true’ prevalence of 50-60% for intestinal schistosomiasis alone. Taken as a whole with urine-filtration and antigen methods, this location should be considered a high-risk environment for schistosomiasis. However, amongst the mothers, general awareness of schistosomiasis was very low which likely contributes to behaviour that continues to sustain high transmission; more than half of the women daily bathed or washed clothes in environmental water. This also influenced childhood exposure; PSAC accompanied their mothers at the waters’ edge, infant bathing was directly witnessed during snail surveys and 1 in 5 PSAC were reported to be bathed daily in this water (Figure 6). Similar practices have been reported elsewhere [16].

Persistent infection with schistosomiasis typically gives rise to chronic multi-organ damage through immunopathological lesions to trapped eggs [1]. Even light egg-patent infections, and more recently sub-egg-patent infections, are considered detrimental to well-being, especially in younger children who are at a more vulnerable stage in growth and development [49,50]. By contrast, microhaematuria and albuminuria are very clear indicators of lower urinary tract disease and the prognosis of such individuals without access to treatment likely to be poor, with clear detrimental clinical outcomes downstream [51]. As shown in Table 1 and Figure 6, elevated levels of albuminuria were strongly associated with *S. haematobium* infection in both mothers and PSAC [33,52]. We recommend that future study of disease sequelae with ultrasonography is a priority, and would add to other evidence accrued in Mali and Zimbabwe of severe urinary tract disease [15,52,53].

### Observations on transmission

Active cases of egg-patent *S. mansoni* infections were found in 21.5% of mothers in the first 6 villages and overall, 24.9% of mothers and 9.1% of children had evidence of intestinal schistosomiasis by urine CCA-dipsticks. The occurrence of intestinal schistosomiasis in this area is intriguing. It has previously been assumed that this area is only endemic for urogenital schistosomiasis [42,54,55]. Whilst snail surveys confirmed the local presence of *B. globosus*, no population of *Biomphalaria* was found so the current transmission risk of *S. mansoni* could be considered low. DNA barcoding showed that the *S. mansoni* found in Chikhwawa has an identical DNA barcode to other inspected isolates from more northern parts of Malawi together with other isolates from this region of continental southern Africa, i.e. *S. mansoni* Groups IV & V in Zambia [41].

To explain this unusual epidemiology, two potential hypotheses are feasible which are not mutually exclusive. First, that infections of intestinal schistosomiasis were not locally acquired and were contracted elsewhere, e.g. around Blantyre, which might explain why mothers were at greater risk owing to longer peripatetic history than their child. Second, that local transmission of intestinal schistosomiasis occurs but intermittently through time, e.g. shortly after periods of prior flooding. Populations of *Biomphalaria* are typically restricted to parts of Africa where thermal maxima are not as extreme [2] but could potentially colonize Chikhwawa when washed-in during local flooding. Nevertheless the prevalence of schistosomiasis reported here is much higher than that reported previously by Bowie *et al.*[54] in surveys of SAC using standard parasitological sampling.

As *B. globosus* is an intermediate snail host of *S. haematobium* in Malawi [43,46,56,57], it was unsurprising that local prevalence of urogenital schistosomiasis was high close to locations where these were found, see Figure 1. DNA barcoding revealed the presence of one of the two groups of *S. haematobium*, i.e. Group I, in the sample and an inferred absence of Group II types [40]. Group I types are widespread across Africa whereas Group II is restricted to coastal East African and associated islands in the Indian Ocean. Although atypical eggs were found which might be considered to be *Schistosoma leiperi*, see Figure 6, DNA barcoding suggested that these were of *S. haematobium* origin with typical mitochondrial sequences, which suggesting that these putative hybrids have retained a maternal mitochondrial lineage from an ancestral *S. haematobium* parental stock [40]. Nonetheless, with the occurrence of atypical eggs, zoonotic transmission should be considered further.

## Conclusion

We have demonstrated a significant but previously overlooked burden of urogenital schistosomiasis amongst PSAC and their mothers in Chikhwawa district, and occurrence of co-infection with intestinal schistosomiasis. To conclude, greater surveillance of schistosomiasis in PSAC and their mothers throughout Malawi is advised alongside much needed interventions administering PZQ to them. Further investigation of the extent of putative zoonotic transmission of urogenital schistosomiasis and local transmission of intestinal schistosomiasis in this southern part of Malawi is recommended.

## Competing interests

The authors declare that they have no competing interests.

## Authors’ contributions

HP, DJT, DGL and JRS jointly conceived of the project and obtained funding. Fieldwork was undertaken by HP, AN, KM and JRS. Data input and analysis was conducted by HP, DJT and MS. Schistosome and snail samples were collected by JRS and analyzed in the laboratory by MB and JRS. Initial draft of the manuscript was prepared by HP with DJT. All authors read and approved the final version of the manuscript.
